# The Association between the Hematocrit at Admission and Preoperative Deep Venous Thrombosis in Hip Fractures in Older People: A Retrospective Analysis

**DOI:** 10.3390/jcm12010353

**Published:** 2023-01-02

**Authors:** Dong-Yang Li, Dong-Xing Lu, Ting Yan, Kai-Yuan Zhang, Bin-Fei Zhang, Yu-Min Zhang

**Affiliations:** 1 Department of Orthopedic Trauma, Honghui Hospital, Xi’an Jiaotong University, Xi’an 710054, China; 2 Department of Ultrasound Medicine, Honghui Hospital, Xi’an Jiaotong University, Xi’an 710054, China; 3 Department of Joint Surgery, Honghui Hospital, Xi’an Jiaotong University, Xi’an 710054, China

**Keywords:** hematocrit, hip fracture, DVT, logistic regression, retrospective

## Abstract

Hematocrit, a commonly used hematological indicator, is a simple and easily applicable test. As a marker of anisocytosis and anemia, it indicates the percentage of blood cells per unit volume of whole blood. This study aimed to evaluate the association between the level of the hematocrit at admission and preoperative deep vein thrombosis (DVT) in hip fractures of older people. We collected the demographic and clinical characteristics of patients with geriatric hip fractures between 1 January 2015, and 30 September 2019, at the largest trauma center in northwestern China. Doppler ultrasonography was used to diagnose DVT. The correlation between hematocrit levels at admission and preoperative DVT was assessed using linear and nonlinear multivariate logistic regression, according to the adjusted model. All analyzes were performed using EmpowerStats and R software. In total, 1840 patients were included in this study, of which 587 patients (32%) had preoperative DVT. The mean hematocrit level was 34.44 ± 5.64 vol%. Linear multivariate logistic regression models showed that admission hematocrit levels were associated with preoperative DVT (OR = 0.97, 95% CI: 0.95–0.99; *p* = 0.0019) after adjustment for confounding factors. However, the linear association was unstable, and nonlinearity was identified. An admission hematocrit level of 33.5 vol% was an inflection point for the prediction. Admission hematocrit levels <33.5 vol% were not associated with preoperative DVT (OR = 1.00, 95% CI: 0.97–1.04, *p* = 0.8230), whereas admission hematocrit levels >33.5 vol% were associated with preoperative DVT (OR = 0.94, 95% CI: 25 0.91–0.97, *p* = 0.0006). Hematocrit levels at admission were nonlinearly associated with preoperative DVT, and hematocrit at admission was a risk factor for preoperative DVT. However, the severity of a low hematocrit was not associated with preoperative DVT when the hematocrit was <33.5 vol%.

## 1. Introduction

As the main type of osteoporotic fracture, hip fractures have a high incidence in the older population [1]. The number of hip fractures worldwide is estimated to reach 4.5 million by 2050 [2]. With the aging population and longer life expectancy, patients with hip fractures are a major challenge for the healthcare system and society due to poor prognosis [3,4,5]. Patients with hip fractures often have other diseases and are in poor physical condition. Therefore, older adults are at risk for prolonged bed rest after hip fractures.

Kaperonis et al. found that 5-day bed rest in a normal person results in sluggish blood flow, increased red blood cell aggregation, and increased blood viscosity, which can induce deep vein thrombosis (DVT) [6]. DVT is common in older adults with hip fractures due to trauma, immobilization, advanced age, and comorbidities [7,8]. The reported prevalence of perioperative DVT after hip fracture ranges from 11.1 to 29.4% [9,10].

For patients at high risk of thrombosis, proactive measures should be taken in time to prevent and treat DVT. Otherwise, it can lead to chronic pain, and secondary varicose, even fatal, pulmonary embolism (PE) can occur, which seriously affects the quality of life and increases the hospitalization costs [11,12]. There has been considerable research on the prevention of DVT, but optimal preventive measures have not been established. Rivaroxaban or low-molecular-weight heparin (LMWH) is a treatment for DVT prophylaxis [13,14]. However, it has not been particularly effective. The incidence of DVT is still 20–30% [10,15]. Therefore, it is necessary to analyze in depth the risk factors for perioperative DVT, which may help prevent the further development of this complication.

The hematocrit is a commonly used hematological indicator as a marker for anisocytosis and anemia, and indicates the percentage of red blood cells per unit volume of whole blood [16]. It is one of the main determinants of blood viscosity, and an increased hematocrit is associated with increased blood viscosity, decreased venous return, and increased exposure of endothelial cells to platelets and coagulation factors [17]. Therefore, subjects with Hct levels above the normal range are theoretically susceptible to DVT. Previous studies have shown a correlation between Hct level and DVT. However, the relationship between hematocrit and DVT is not sufficiently detailed and remains controversial [18,19,20]. Data from previous studies were based on the general population rather than on patients with fractures. Regarding hip fractures in older adults, evidence on the relationship between the hematocrit level at admission and preoperative DVT is lacking. Therefore, it is necessary to build a reliable model to understand the association between Hct levels at admission and DVT or to predict the prognosis.

This study aimed to evaluate the association between the level of the hematocrit at admission and preoperative DVT in older adults with hip fractures. We hypothesized that there is a linear or nonlinear association between hematocrit level at admission and preoperative DVT, which would explain the effect of hematocrit level at admission on preoperative DVT and provide a target for prevention.

## 2. Materials and Methods

### 2.1. Ethics Statement

We recruited older adults with a hip fracture between 1 January 2015, and 30 September 2019, at the largest trauma center in Northwest China.

The Ethics Committee of our hospital (No. 202201009) approved this retrospective study. All human procedures were performed in accordance with the Declaration of Helsinki of 1964 and its subsequent amendments. The study was conducted according to the STROCSS 2021 guidelines [21].

### 2.2. Inclusion and Exclusion Criteria

The demographic and clinical data of the patients were obtained from their original medical records. The inclusion criteria were as follows: (1) age ≥65 years; (2) diagnosis by X-ray or computed tomography of femoral neck or intertrochanteric or subtrochanteric fracture; and (3) patients receiving surgical or conservative treatment in the hospital. The exclusion criteria were as follows: patients for whom clinical data in the hospital were unavailable.

### 2.3. Hospital Treatment

The patients were examined using blood tests and ultrasonography to prepare for surgery. Prophylaxis for deep vein thrombosis was initiated at admission. A mechanical pressure pump (20 min, twice daily) was used to promote blood reflux. Furthermore, for patients without contraindications, LMWH was subcutaneously injected according to guidelines to prevent DVT. Anticoagulant therapy was discontinued 12 h before the operation and resumed 24 h after the operation. Blood samples were collected at the time of admission (2 h after admission).

### 2.4. DVT Diagnosis

**Figure 1:**
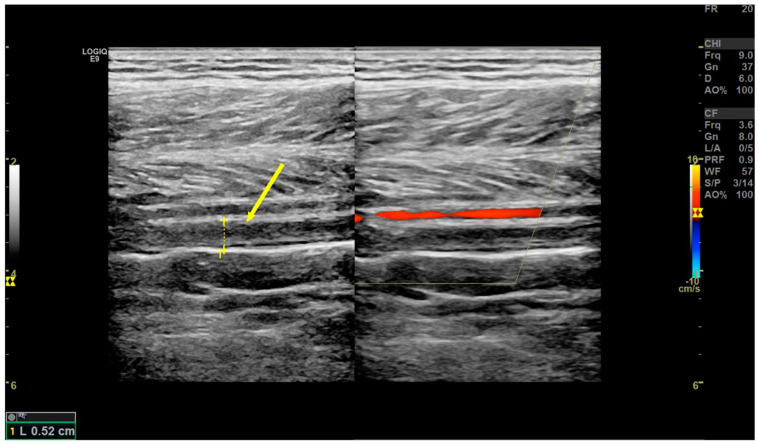
DVT in Doppler ultrasonography (yellow arrow).

According to Chinese guidelines for the prevention of venous thromboembolism in orthopedic surgery, color Doppler ultrasound was used to detect DVT. Vascular ultrasonography was performed using a bedside machine by three trained operators. The diagnostic criterion for fresh thrombosis was the presence of a constant intraluminal filling defect [22], as shown in Figure 1. Anticoagulation regimens were guided by hospital consultations during vascular surgery. If required, an inferior vena cava filter was used to prevent fatal pulmonary embolism.

### 2.5. Endpoint Events

The endpoint event in this study was preoperative DVT.

### 2.6. Variables

In this study, the following variables were collected: hematocrit level, age, sex, occupation, history of allergy, injury mechanism, fracture classification, hypertension, diabetes, coronary heart disease, arrhythmia, hemorrhagic stroke, ischemic stroke, cancer, associated injuries, dementia, chronic obstructive pulmonary disease (COPD), hepatitis, gastritis, age−adjusted Charlson comorbidity index (aCCI), and time from injury to admission.

The dependent variable was preoperative DVT, and the independent variable was the level of the hematocrit. Other variables were confounding factors.

### 2.7. Statistics Analysis

Descriptive statistical analyzes were performed using standard reporting methods. Continuous variables are reported as mean ± standard deviation (normally distributed data) or median (interquartile range) (nonnormally distributed data). Categorical variables were reported as percentages. Chi−square (categorical variables), one−way ANOVA (normal distribution), or Kruskal–Wallis H tests (skewed distribution) were used to detect differences among different levels of the hematocrit at admission.

We analyzed the association between Hct level and preoperative DVT. Univariate and multivariate binary logistic regression models were used to test the association between Hct levels and preoperative DVT using three distinct models. Model 1: No covariates are adjusted. Model 2 was a minimally adjusted model, adjusted only for sociodemographic covariates. Model 3 was fully adjusted for all covariates. We performed a sensitivity analysis to verify the robustness of the results. We converted admission hematocrit into a categorical variable according to the quintiles, calculated *p* for the trend to verify the results of admission hematocrit as a continuous variable, and examined the possibility of nonlinearity (Q1–Q5 groups).

To account for the nonlinear relationship between hematocrit and preoperative DVT, we also used a generalized additive model and smooth curve fitting (penalized spline method) to address nonlinearity. If nonlinearity was detected, we first calculated the inflection point using a recursive algorithm and then constructed a two−piece logistic proportional hazard regression model for each side of the inflection point.

All analyzes were performed using the statistical software packages R (http://www.R-project.org, R Foundation, Vienna, Austria) (accessed on 25 September 2022) and EmpowerStats (http://www.empowerstats.com, X&Y Solutions Inc., Boston, MA, USA) (accessed on 25 September 2022). Statistical significance was established by a two−sided *p*−value, where *p* < 0.05 (two-sided) was considered to be statistically significant.

## 3. Results

### 3.1. Patient Characteristics

**Figure 2:**
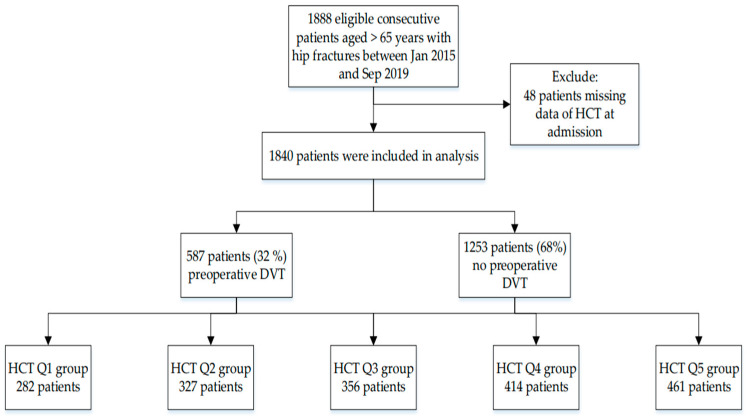
Study flow diagram.

Of the 1881 participants with hip fractures between January 2015 and September 2019, 48 patients were excluded from this study due to missing HCT data at admission. A total of 1840 patients met the study criteria and were enrolled in our study. A flowchart is shown in Figure 2. Hematocrit levels were divided into five groups. The average admission hematocrit of all patients was 34.44 ± 5.64 vol% (Q1 group: 25.35 ± 2.96 vol%, Q2 group: 30.62 ± 1.08 vol%, Q3 group: 33.75 ± 0.83 vol%, Q4 group: 36.61 ± 0.88 vol%, and Q5 group: 41.29 ± 2.67 vol%). A total of 587 patients (32%) had preoperative DVT ((Q1 group: 95 (33.69%); Q2 group: 120 (36.70%); Q3 group: 135 (37.92%); Q4 group: 125 (30.19%); and Q5 group: 112 (24.30%)). Eight patients had pulmonary embolism, and two died after the operation due to coronary heart disease (CHD).

Table 1 lists the demographic and clinical characteristics of all 1840 patients, including comorbidities, factors associated with injuries, and hematocrit levels at admission.

### 3.2. Univariate Analysis

To identify possible confounders and the relationship between admission hematocrit level and preoperative DVT, we performed a univariate analysis (Table 2). According to the criteria of *p* < 0.1, the following variables were considered in the multivariate logistic regression: sex, dementia, and multiple injuries.

### 3.3. Multivariate Analysis between Admission Hematocrit Level and Preoperative DVT

We used three models (Table 3) to correlate hematocrit levels and preoperative DVT. When the hematocrit level was a continuous variable, linear regression was observed. The fully adjusted model showed a preoperative decrease in the risk of DVT of 3% (OR = 0.97, 95% CI: 0.95–0.99, *p* = 0.0019) when hematocrit levels increased by 1% after controlling for confounders. When hematocrit levels were used as a categorical variable, we found statistically significant differences in the hematocrit level groups of the three models (*p* < 0.05). Compared with the hematocrit Q1 group, the hematocrit Q5 group could decrease the risk of preoperative DVT by 31% (OR = 0.69, 95% CI: 0.50–0.97, *p* = 0.0304). However, there were no statistically significant differences among the Q2–Q4 hematocrit groups and the Q1 group. In addition, the *p* for the trend showed *p* < 0.05 in the three models. This instability indicates a nonlinear correlation.

### 3.4. Curve Fitting and Analysis of Threshold Effect

**Figure 3:**
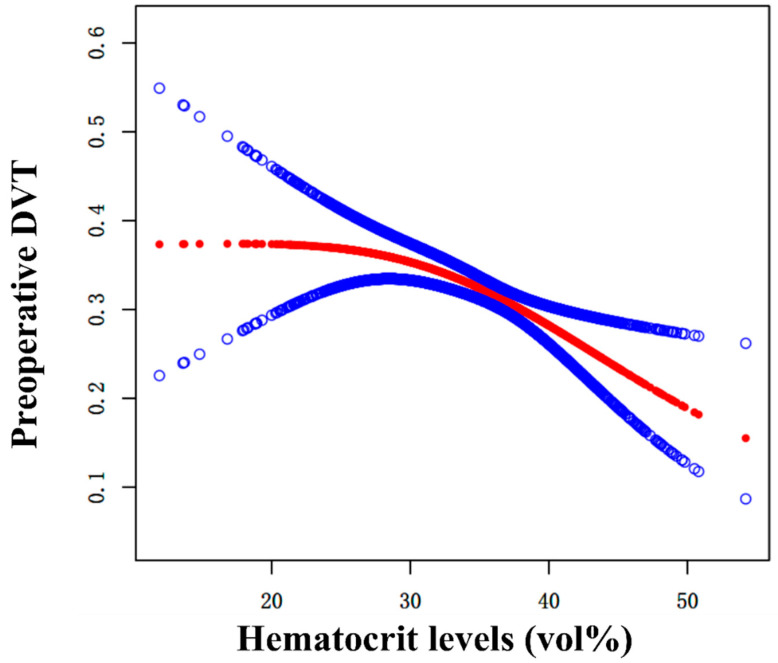
Curve fitting between admission hematocrit levels and preoperative DVT. Adjusted for sex, dementia, and multiple injuries.

As shown in Figure 3, after adjusting for confounders, we fit a curve to explain the association between Hct levels at admission and preoperative DVT. We compared two fitting models to explain this association (Table 4). Interestingly, an inflection point was observed. Admission hematocrit levels of >33.5 vol% were associated with preoperative DVT (OR = 0.94, 95% CI: 0.91–0.97, *p* = 0.0006). At admission hematocrit levels of <33.5 vol%, there was no statistically significant correlation between preoperative DVT and admission hematocrit levels (OR = 0.94, 95% CI: 0.91–0.97, *p* = 0.0006).

## 4. Discussion

Our study shows that the level of hematocrit at admission is a strong predictor of preoperative DVT in older adults with hip fractures. Specifically, hematocrit levels at admission were nonlinearly associated with preoperative DVT. A hematocrit level of 33.5 vol% was the inflection point in the saturation effect. When the hematocrit level was <33.5 vol%, the hematocrit level at admission was not a potential risk factor for preoperative DVT (OR = 1.00), and the severity of low hematocrit was not associated with preoperative DVT. When the hematocrit level was >33.5 vol%, for each unit increase in hematocrit, the risk of preoperative DVT decreased by 6% (OR = 0.94). Therefore, a hematocrit level of 33.5 vol% is a useful indicator to predict preoperative DVT in older patients with hip fractures. In clinical practice, these findings can be used to identify high-risk patients who may benefit from specialized care.

Older adults with hip fractures are prone to DVT events due to advanced age, comorbidities (hypertension, heart disease, peripheral vascular disease, cerebrovascular disease), or risk factors (trauma, surgery, limb immobilization), further aggravating their poor prognosis. According to previous studies, the incidence of preoperative DVT in patients with hip fracture is 8–34.9%, and the incidence of DVT in patients with delayed surgery can even reach 62% [9,23,24]. A meta-analysis of 2022 analyzed 9823 patients and found that the incidence of preoperative DVT in elderly patients with hip fractures was 16.6%. Age, sex, BMI, low hemoglobin level, time from injury to admission, time from injury to surgery, type of hip fracture, CHD, dementia, pulmonary disease, kidney disease, smoking, fibrinogen, C-reactive protein, and albumin were considered independent risk factors for DVT [25].

Patients with polycythemia vera have been shown to be associated with an increased risk of DVT [26]. A Mendelian randomization study showed that a polygenic risk score for hemoglobin concentration was positively associated with venous thromboembolism risk in the general population [27]. Therefore, the hypothesis that detection of Hct levels has a predictive effect on the occurrence of DVT in elderly hip fractures has strong biological plausibility. The relationship between hematocrit levels and DVT incidence has previously been studied. A prospective study in Norway evaluating 26,108 adults showed that subjects with a hematocrit in the upper 20th percentile had a 1.5-fold higher risk of total DVT compared with subjects with a hematocrit in the lowest 40th percentile [19]. A case–control trial by Vayá et al. found that the proportion of subjects with a hematocrit greater than 45% was significantly higher in patients with DVT than in healthy controls [28]. A population-based cohort study from Denmark found a U-shaped association between Hct and VTE, but the association was not statistically significant [29]. A 2020 study showed that high levels of hematocrit and hemoglobin are associated with an increased long-term risk of VTE [18]. However, in a population-based longitudinal investigation of the etiology of thromboembolism, no significant association was reported between the hematocrit and the incidence of VTE [30]. An earlier case–control study also found no independent relationship between Hct and VTE [31]. Based on the above controversy, it remains to be further investigated whether hematocrit is the real cause of DVT or an innocent interloper, that is, whether the relationship between the two is causal or whether the relationship is confounded by other confounding factors [32]. 

Previous studies have been based on the general population. Results based on the general population are generally considered to have limited significance for specific populations. To our knowledge, this is the first study to investigate the relationship between hematocrit levels at admission and preoperative DVT in geriatric hip fractures. The prevalence of preoperative DVT in these patients is high (32%). We believe that previous studies may have underestimated the incidence of DVT. It is possible that the symptoms of hip fracture can mask the clinical signs and symptoms of DVT [33]. In this study, we established an association using curve fitting and found a saturation point and, therefore, a meaningful prediction point. Our study showed that hematocrit levels <33.5 vol% were not associated with preoperative DVT, whereas hematocrit levels at admission of >33.5 vol% were associated with preoperative DVT. Furthermore, according to the current anemia criteria, our study supports that anemia is a risk factor for developing DVT, and higher levels of HCT are associated with a lower risk of DVT.

To avoid the impact of COVID-19 on patient admission [34] and for a more accurate assessment of the relationship between hematocrit levels at admission and preoperative DVT, we performed linear regression on the adjusted model and comprehensively considered the variables that needed to be adjusted. Factors with *p* < 0.1 in the univariate analysis and factors included in previous studies were considered. Specifically, we used a sensitivity analysis of the trend test in the linear model. In addition, we considered the association of the curve and found a clinical saturation effect and an inflection point. Curve fitting was more suitable than linear fitting to explain the association between admission hematocrit levels and preoperative DVT.

Despite the large sample size and the many methods used to explain the relationship between variables and preoperative DVT, this study had several limitations. First, as with every other multivariate analysis, we were unable to include all confounding factors. Therefore, the residual confounding factors remained. Second, due to the limitations of the retrospective study design, we could not assess the progression of Hct levels over time. Third, our study was a single center study; all samples were from the same hospital, and hematocrit levels were strongly associated with region and ethnicity [35]. Therefore, these results should be interpreted with caution and the inference points for other ethnicities should be redefined.

In conclusion, hematocrit levels at admission were nonlinearly associated with preoperative DVT, and the hematocrit level at admission was a risk indicator of preoperative DVT. However, the severity of low hematocrit was not associated with preoperative DVT when the hematocrit was <33.5 vol%.

## Figures and Tables

**Table 1:** The demographic and clinical characteristics of the patients (N = 1840).

Hematocrit Quintiles	Q1 (n = 282)	Q2 (n = 327)	Q3 (n = 356)	Q4 (n = 414)	Q5 (n = 461)	*p*-Value	*p*-Value *
Age (year)	82.61 ± 6.32	80.53 ± 6.90	79.62 ± 6.30	78.66 ± 6.57	77.14 ± 6.98	<0.001	<0.001
Sex						<0.001	-
Male	55 (19.50%)	89 (27.22%)	94 (26.40%)	109 (26.33%)	211 (45.77%)		
Female	227 (80.50%)	238 (72.78%)	262 (73.60%)	305 (73.67%)	250 (54.23%)		
Injury mechanism						0.527	-
Falling	272 (96.45%)	317 (96.94%)	339 (95.22%)	404 (97.58%)	441 (95.66%)		
Traffic accident	7 (2.48%)	7 (2.14%)	14 (3.93%)	10 (2.42%)	15 (3.25%)		
Other	3 (1.06%)	3 (0.92%)	3 (0.84%)	0 (0.00%)	5 (1.08%)		
Fracture classification						<0.001	-
Intertrochanteric fracture	228 (80.85%)	261 (79.82%)	236 (66.29%)	199 (48.07%)	182 (39.48%)		
Femoral neck fracture	45 (15.96%)	59 (18.04%)	113 (31.74%)	208 (50.24%)	276 (59.87%)		
Subtrochanteric fracture	9 (3.19%)	7 (2.14%)	7 (1.97%)	7 (1.69%)	3 (0.65%)		
Hypertension	123 (43.62%)	160 (48.93%)	184 (51.69%)	203 (49.03%)	254 (55.10%)	0.039	-
Diabetes	45 (15.96%)	60 (18.35%)	82 (23.03%)	82 (19.81%)	98 (21.26%)	0.202	-
CHD	142 (50.35%)	163 (49.85%)	193 (54.21%)	198 (47.83%)	254 (55.10%)	0.187	-
Arrhythmia	98 (34.75%)	97 (29.66%)	107 (30.06%)	113 (27.29%)	168 (36.44%)	0.029	-
Hemorrhagic stroke	5 (1.77%)	6 (1.83%)	6 (1.69%)	5 (1.21%)	10 (2.17%)	0.877	-
Ischemic stroke	82 (29.08%)	104 (31.80%)	111 (31.18%)	114 (27.54%)	169 (36.66%)	0.05	-
Cancer	7 (2.48%)	13 (3.98%)	9 (2.53%)	11 (2.66%)	8 (1.74%)	0.431	-
Dementia	20 (7.09%)	8 (2.45%)	10 (2.81%)	18 (4.35%)	15 (3.25%)	0.022	-
Multiple injuries	36 (12.77%)	31 (9.48%)	23 (6.46%)	23 (5.56%)	18 (3.90%)	<0.001	-
COPD	17 (6.03%)	18 (5.50%)	21 (5.90%)	19 (4.59%)	31 (6.72%)	0.75	-
Hepatitis	15 (5.32%)	6 (1.83%)	10 (2.81%)	11 (2.66%)	13 (2.82%)	0.135	-
Gastritis	6 (2.13%)	4 (1.22%)	9 (2.53%)	5 (1.21%)	3 (0.65%)	0.194	-
Time to admission (h)	71.83 ± 122.56	80.71 ± 159.64	86.29 ± 263.66	108.05 ± 407.03	70.30 ± 172.10	0.22	<0.001
aCCI	4.49 ± 0.99	4.28 ± 1.05	4.27 ± 1.16	4.11 ± 1.17	4.05 ± 1.07	<0.001	<0.001
Hematocrit	25.35 ± 2.96 (11.90-28.50)	30.62 ± 1.08 (28.60–32.20)	33.75 ± 0.83 (32.30–35.10)	36.61 ± 0.88 (35.20–38.20)	41.29 ± 2.67 (38.30–54.20)	<0.001	<0.001
DVT	95 (33.69%)	120 (36.70%)	135 (37.92%)	125 (30.19%)	112 (24.30%)	<0.001	-

Mean ± SD/N (%)*. p*-value *: For continuous variables, we used the Kruskal–Wallis rank-sum test, and Fisher’s exact probability test was used for count variables with a theoretical number <10.

**Table 2:** Effects of factors on preoperative DVT measured by univariate analysis (N = 1840).

	Statistics	OR (95% CI)	*p*-Value
Age (year)	79.40 ± 6.88	1.00 (0.99, 1.02)	0.7005
Sex			
Male	558 (30.33%)	1	
Female	1282 (69.67%)	1.26 (1.01, 1.56)	0.0388
Injury mechanism			
Falling	1773 (96.36%)	1	
Traffic accident	53 (2.88%)	1.11 (0.62, 1.98)	0.7206
Other	14 (0.76%)	2.88 (0.99, 8.34)	0.0511
Fracture classification			
Intertrochanteric fracture	1106 (60.11%)	1	
Femoral neck fracture	701 (38.10%)	0.68 (0.56, 0.84)	0.0004
Subtrochanteric fracture	33 (1.79%)	1.77 (0.88, 3.54)	0.1069
Time to admission (h)	83.97 ± 255.05	1.00 (1.00, 1.00)	0.2535
Hypertension			
No	916 (49.78%)	1	
Yes	924 (50.22%)	1.13 (0.93, 1.38)	0.2215
Diabetes			
No	1473 (80.05%)	1	
Yes	367 (19.95%)	0.95 (0.74, 1.22)	0.6998
CHD			
No	890 (48.37%)	1	
Yes	950 (51.63%)	1.07 (0.88, 1.30)	0.488
Arrhythmia			
No	1257 (68.32%)	1	
Yes	583 (31.68%)	1.07 (0.87, 1.32)	0.5182
Hemorrhagic stroke			
No	1808 (98.26%)	1	
Yes	32 (1.74%)	1.68 (0.83, 3.39)	0.1512
Ischemic stroke			
No	1260 (68.48%)	1	
Yes	580 (31.52%)	0.86 (0.69, 1.06)	0.1608
Cancer			
No	1792 (97.39%)	1	
Yes	48 (2.61%)	1.18 (0.65, 2.14)	0.5969
Dementia			
No	1769 (96.14%)	1	
Yes	71 (3.86%)	1.59 (0.98, 2.58)	0.0583
Multiple injuries			
No	1709 (92.88%)	1	
Yes	131 (7.12%)	1.60 (1.12, 2.30)	0.0108
COPD			
No	1734 (94.24%)	1	
Yes	106 (5.76%)	0.88 (0.57, 1.35)	0.5457
Hepatitis			
No	1785 (97.01%)	1	
Yes	55 (2.99%)	0.65 (0.35, 1.23)	0.1849
Gastritis			
No	1813 (98.53%)	1	
Yes	27 (1.47%)	0.48 (0.18, 1.28)	0.1413
aCCI	4.21 ± 1.10	0.99 (0.91, 1.09)	0.8784
Hematocrit	34.44 ± 5.64	0.97 (0.95, 0.98)	0.0002

**Table 3:** Multivariate results by logistic regression (N = 1840).

Exposure	Non-Adjusted Model	Minimally-Adjusted Model	Fully-Adjusted Model
Hematocrit	0.97 (0.95, 0.98) 0.0002	0.97 (0.95, 0.99) 0.0006	0.97 (0.95, 0.99) 0.0019
Hematocrit quintiles			
Q1	1	1	1
Q2	1.14 (0.82, 1.59) 0.4385	1.15 (0.83, 1.61) 0.3996	1.20 (0.85, 1.68) 0.2943
Q3	1.20 (0.87, 1.67) 0.2689	1.22 (0.88, 1.69) 0.2431	1.27 (0.92, 1.77) 0.1494
Q4	0.85 (0.62, 1.18) 0.3305	0.86 (0.62, 1.19) 0.3626	0.90 (0.65, 1.24) 0.5175
Q5	0.63 (0.46, 0.88) 0.0058	0.66 (0.47, 0.91) 0.0129	0.69 (0.50, 0.97) 0.0304
*P* for trend	0.0002	0.0007	0.002

Data in table: OR (95% CI)**,** *p*-value. Outcome variable: preoperative DVT. Exposure variable: hematocrit level at admission. Minimally-adjusted model: adjust for sex. Fully-adjusted model: adjust for sex, dementia, and multiple injuries.

**Table 4:** Nonlinearity of admission hematocrit levels versus preoperative DVT (N = 1840).

Outcome:	OR (95% CI) *p*-Value
Fitting model by standard linear regression	0.97 (0.95, 0.99) 0.0019
Fitting model by two-piecewise linear regression	
Inflection point	33.5 vol%
<33.5 vol%	1.00 (0.97, 1.04) 0.8230
>33.5 vol%	0.94 (0.91, 0.97) 0.0006
*P* for log-likelihood ratio test	0.029

Adjusted for sex, dementia, and multiple injuries.

## Data Availability

Data were obtained from the Xi’an Honghui Hospital. According to relevant regulations, the data could not be shared but can be requested from the corresponding author.

## Abbreviations

DVT	deep venous thrombosis
OR	odds ratio
CI	confidence interval
LMWH	low-molecular-weight heparin
CHD	coronary heart disease
COPD	chronic obstructive pulmonary disease
aCCI	age-adjusted Charlson comorbidity index

## References

[1] Gullberg B., Johnell O., Kanis J. (1997). World-wide Projections for Hip Fracture. Osteoporos. Int..

[2] Veronese N., Maggi S. (2018). Epidemiology and social costs of hip fracture. Injury.

[3] Peeters C.M.M., Visser E., Van de Ree C.L.P., Gosens T., Den Oudsten B.L., De Vries J. (2016). Quality of life after hip fracture in the elderly: A systematic literature review. Injury.

[4] Koso R.E., Sheets C., Richardson W.J., Galanos A.N. (2017). Hip Fracture in the Elderly Patients: A Sentinel Event. Am. J. Hosp. Palliat. Med..

[5] Meinberg E., Ward D., Herring M., Miclau T. (2020). Hospital-based Hip fracture programs: Clinical need and effectiveness. Injury.

[6] Kaperonis A.A., Michelsen C.B., Askanazi J., Kinney J.M., Chien S. (1988). Effects of Total Hip Replacement and Bed Rest on Blood Rheology and Red Cell Metabolism. J. Trauma Inj. Infect. Crit. Care.

[7] Cho Y.-H., Byun Y.-S., Jeong D.-G., Han I.-H., Park Y.-B. (2015). Preoperative Incidence of Deep Vein Thrombosis after Hip Fractures in Korean. Clin. Orthop. Surg..

[8] Luksameearunothai K., Sa-Ngasoongsong P., Kulachote N., Thamyongkit S., Fuangfa P., Chanplakorn P., Woratanarat P., Suphachatwong C. (2017). Usefulness of clinical predictors for preoperative screening of deep vein thrombosis in hip fractures. BMC Musculoskelet. Disord..

[9] Shin W., Woo S., Lee S., Lee J., Kim C., Suh K. (2016). Preoperative Prevalence of and Risk Factors for Venous Thromboembolism in Patients with a Hip Fracture: An Indirect Multidetector CT Venography Study. J. Bone Jt. Surg. Am..

[10] Song K., Yao Y., Rong Z., Shen Y., Zheng M., Jiang Q. (2016). The preoperative incidence of deep vein thrombosis (DVT) and its correlation with postoperative DVT in patients undergoing elective surgery for femoral neck fractures. Arch. Orthop. Trauma Surg..

[11] Brill J.B., Badiee J., Zander A.L., Wallace J.D., Lewis P.R., Sise M.J., Bansal V., Shackford S.R. (2017). The rate of deep vein thrombosis doubles in trauma patients with hypercoagulable thromboelastography. J. Trauma Inj. Infect. Crit. Care.

[12] Ruskin K. (2018). Deep vein thrombosis and venous thromboembolism in trauma. Curr. Opin. Anaesthesiol..

[13] Di Nisio M., van Es N., Buller H. (2016). Deep vein thrombosis and pulmonary embolism. Lancet.

[14] Long A., Zhang L., Zhang Y., Jiang B., Mao Z., Li H., Zhang S., Xie Z., Tang P. (2014). Efficacy and safety of rivaroxaban versus low-molecular-weight heparin therapy in patients with lower limb fractures. J. Thromb. Thrombolysis.

[15] Calder J.D.F., Freeman R., Domeij-Arverud E., Van Dijk C.N., Ackermann P. (2016). Meta-analysis and suggested guidelines for prevention of venous thromboembolism (VTE) in foot and ankle surgery. Knee Surg. Sport. Traumatol. Arthrosc..

[16] Yavorkovsky L.L. (2021). Mean corpuscular volume, hematocrit and polycythemia. Hematology.

[17] Lowe G.D.O., Lee A.J., Rumley A., Price J.F., Fowkes F.G.R. (1997). Blood viscosity and risk of cardiovascular events: The Edinburgh Artery Study. Br. J. Haematol..

[18] Folsom A., Wang W., Parikh R., Lutsey P., Beckman J., Cushman M. (2020). Hematocrit and incidence of venous thromboembolism. Res. Pract. Thromb. Haemost..

[19] Braekkan S.K., Mathiesen E.B., Njølstad I., Wilsgaard T., Hansen J.-B. (2009). Hematocrit and risk of venous thromboembolism in a general population. The Tromso study. Haematologica.

[20] Zhang B., Wang P., Fei C., Shang K., Qu S., Li J., Ke C., Xu X., Yang K., Liu P. (2020). Perioperative Deep Vein Thrombosis in Patients with Lower Extremity Fractures: An Observational Study, Clinical and applied thrombosis/hemostasis. Off. J. Int. Acad. Clin. Appl. Thrombosis/Hemostasis.

[21] Mathew G., Agha R., Albrecht J., Goel P., Mukherjee I., Pai P., D’Cruz A.K., Nixon I.J., Roberto K., Enam S.A. (2021). STROCSS 2021: Strengthening the reporting of cohort, cross-sectional and case-control studies in surgery. Int. J. Surg..

[22] Mantoni M. (2001). Ultrasound of limb veins. Eur. Radiol..

[23] Zhang B.-F., Wei X., Huang H., Wang P.-F., Liu P., Qu S.-W., Li J.-H., Wang H., Cong Y.-X., Zhuang Y. (2018). Deep vein thrombosis in bilateral lower extremities after hip fracture: A retrospective study of 463 patients. Clin. Interv. Aging.

[24] Smith E.B., Parvizi J., Purtill J.J. (2011). Delayed Surgery for Patients with Femur and Hip Fractures—Risk of Deep Venous Thrombosis. J. Trauma Inj. Infect. Crit. Care.

[25] Wang T., Guo J., Long Y., Yin Y., Hou Z. (2022). Risk factors for preoperative deep venous thrombosis in hip fracture patients: A meta-analysis, Journal of orthopaedics and traumatology. Off. J. Ital. Soc. Orthop. Traumatol..

[26] Griesshammer M., Kiladjian J.-J., Besses C. (2019). Thromboembolic events in polycythemia vera. Ann. Hematol..

[27] Richardson T.G., Harrison S., Hemani G., Smith G.D. (2019). An atlas of polygenic risk score associations to highlight putative causal relationships across the human phenome. Elife.

[28] Vayá A., Falcó C., Simó M., Ferrando F., Mira Y., Todolí J., España F., Corella L. (2007). Influence of lipids and obesity on haemorheological parameters in patients with deep vein thrombosis. Thromb. Haemost..

[29] Warny M., Helby J., Birgens H.S., Bojesen S.E., Nordestgaard B.G. (2019). Arterial and venous thrombosis by high platelet count and high hematocrit: 108 521 individuals from the Copenhagen General Population Study. J. Thromb. Haemost..

[30] Tsai A., Cushman M., Rosamond W., Heckbert S., Polak J., Folsom A. (2002). Cardiovascular risk factors and venous thromboembolism incidence: The longitudinal investigation of thromboembolism etiology. Arch. Intern. Med..

[31] Vayá A., Mira Y., Martínez M., Villa P., Ferrando F., Estellés A., Corella L., Aznar J. (2002). Biological risk factors for deep vein trombosis. Clin. Hemorheol. Microcirc..

[32] Schreijer A.J., Reitsma P.H., Cannegieter S.C. (2010). High hematocrit as a risk factor for venous thrombosis. Cause or innocent bystander?. Haematologica.

[33] Bengoa F., Vicencio G., Schweitzer D., Lira M., Zamora T., Klaber I. (2020). High prevalence of deep vein thrombosis in elderly hip fracture patients with delayed hospital admission, European journal of trauma and emergency surgery. Off. Publ. Eur. Trauma Soc..

[34] Zhong H., Poeran J., Liu J., Wilson L.A., Memtsoudis S.G. (2021). Hip fracture characteristics and outcomes during COVID-19: A large retrospective national database review. Br. J. Anaesth..

[35] Mondal H., Lotfollahzadeh S. (2022). Hematocrit, StatPearls.

